# Exogenous BMP7 in aortae of rats with chronic uremia ameliorates expression of profibrotic genes, but does not reverse established vascular calcification

**DOI:** 10.1371/journal.pone.0190820

**Published:** 2018-01-05

**Authors:** Eva Gravesen, Maria Lerche Mace, Anders Nordholm, Jacob Hofman-Bang, Keith Hruska, Carsten Haagen Nielsen, Andreas Kjær, Klaus Olgaard, Ewa Lewin

**Affiliations:** 1 Department of Clinical Medicine, Faculty of Health Sciences, University of Copenhagen, Copenhagen, Denmark; 2 Nephrological Department P, Rigshospitalet, Copenhagen, Denmark; 3 Nephrological Department B, Herlev Hospital, Copenhagen, Denmark; 4 Department of Pediatrics, Washington University, St. Louis, MO, United States of America; 5 Department of Biomedical Sciences, Faculty of Health Sciences, University of Copenhagen, Copenhagen, Denmark; 6 Department of Clinical Physiology, Nuclear Medicine & PET and Cluster for Molecular Imaging, Rigshospitalet, University of Copenhagen, Copenhagen, Denmark; University Medical Center Utrecht, NETHERLANDS

## Abstract

Hyperphosphatemia and vascular calcification are frequent complications of chronic renal failure and bone morphogenetic protein 7 (BMP7) has been shown to protect against development of vascular calcification in uremia. The present investigation examined the potential reversibility of established uremic vascular calcification by treatment of uremic rats with BMP7. A control model of isogenic transplantation of a calcified aorta from uremic rats into healthy littermates examined whether normalization of the uremic environment reversed vascular calcification. Uremia and vascular calcification were induced in rats by 5/6 nephrectomy, high phosphate diet and alfacalcidol treatment. After 14 weeks severe vascular calcification was present and rats were allocated to BMP7, vehicle or aorta transplantation. BMP7 treatment caused a significant decrease of plasma phosphate to 1.56 ± 0.17 mmol/L vs 2.06 ± 0.34 mmol/L in the vehicle group even in the setting of uremia and high phosphate diet. Uremia and alfacalcidol resulted in an increase in aortic expression of genes related to fibrosis, osteogenic transformation and extracellular matrix calcification, and the BMP7 treatment resulted in a decrease in the expression of profibrotic genes. The total Ca-content of the aorta was however unchanged both in the abdominal aorta: 1.9 ± 0.6 μg/mg tissue in the vehicle group vs 2.2 ± 0.6 μg/mg tissue in the BMP7 group and in the thoracic aorta: 71 ± 27 μg/mg tissue in the vehicle group vs 54 ± 18 μg/mg tissue in the BMP7 group. Likewise, normalization of the uremic environment by aorta transplantation had no effect on the Ca-content of the calcified aorta: 16.3 ± 0.6 μg/mg tissue pre-transplantation vs 15.9 ± 2.3 μg/mg tissue post-transplantation. Aortic expression of genes directly linked to extracellular matrix calcification was not affected by BMP7 treatment, which hypothetically might explain persistent high Ca-content in established vascular calcification. The present results highlight the importance of preventing the development of vascular calcification in chronic kidney disease. Once established, vascular calcification persists even in the setting when hyperphosphatemia or the uremic milieu is abolished.

## Introduction

Vascular calcification (VC) is a part of the definition of chronic kidney disease-mineral and bone disorder (CKD-MBD) and represents a complex systemic manifestation of chronic kidney insufficiency [1]. VC is influenced by derangements of calcium (Ca) and phosphate (P) homeostasis, dysregulated calcification inhibitors and promoters, and underlying renal osteodystrophy and vascular pathology [1]. The burden of VC is extremely high in CKD [2]. Given the significant morbidity and mortality associated with VC in CKD patients [3;4] it is essential to develop treatments to reverse or slow down this process. Hyperphosphatemia is a common complication of CKD and is associated with increased cardiovascular morbidity and mortality [5]. Increasing plasma P levels, even within the normal range, are correlated with cardiovascular events and mortality in both patients with CKD [6] and in patients with preserved renal function [7]. High P has been shown to induce a phenotypic shift of cells in the vessel wall towards an osteogenic phenotype. *In vitro* evidence suggests that this phenotypic shift is mediated by the sodium-dependent phosphate co-transporter Pit-1 [8].

For a long time it was thought that the calcifications resulted from passive deposition of Ca and P in the vascular tissue. Experimental and clinical studies have later shown that VC is a regulated process akin to bone formation with proteins involved in bone metabolism being expressed in the arterial wall in uremia [9]. The uremic milieu provides extreme calcification stress and calcification of the lamina media is predominant in CKD [10]. In response to injury, contractile vascular smooth muscle cells (VSMC) become proliferative and synthesize factors attempting vessel repair, and in uremia VSMC undergo maladaptive osteochondrocytic differentiation [9;11]. In a previous investigation we used high-throughput RNA sequencing (RNA-seq) for a comprehensive identification of transcriptomes from aortas of normal rats and calcified aortas of uremic rats [12]. The dramatic change in phenotype that occurred during the development of uremic VC was connected to a major shift in the expression profile of genes related to extracellular matrix (ECM) regulation, osteogenic transformation and Wnt modulation.

Bone morphogenetic proteins (BMPs) are widely expressed during mammalian development. In addition to its role in bone formation, BMP7 is a critical developmental and differentiation factor in the kidney [13;14]. In adult kidneys BMP7 expression is retained and BMP7 is considered essential in maintaining tubular epithelial integrity. During various renal diseases epithelial expression of BMP7 is lost [15–17]. Administration of exogenous BMP7 or reactivation of BMP signalling stimulates renal repair and prevents progression of renal disease [18–22]. Systemic BMP7 administration in experimental models of CKD has revealed favourable extra-renal actions [23]. In animal models of CKD-MBD with established secondary hyperparathyroidism, BMP7 administration stimulates bone formation and increases bone mass [24], and in animal models with adynamic bone disease BMP7 restores skeletal remodelling to normal rate [25;26]. A preventive effect of BMP7 on development of VC has been shown in the LDL receptor null mice with superimposed CKD by Davies et al. [27]. BMP7 therapy increased vascular smooth muscle cell (VSMC) differentiation, decreased vascular osteoblastic gene expression and impaired VC. The mechanisms behind this protective effect is not fully known, although evidence from a later study from the same group suggested that the effect at least in part could be mediated by normalization of the CKD-associated bone lesions and correction of hyperphosphatemia [26].

In the present investigation, the effect of administration of exogenous BMP7 on already established uremic vascular calcifications was examined with special focus upon whether BMP7 could reverse the VC and whether BMP7 had an impact on the gene expressions in the uremic vasculature. As a control it was examined whether normalization of the uremic environment could reverse established VC. This was achieved by isogenic transplantation of the calcified aorta from uremic rats into healthy littermates.

## Methods

### Animals

Inbred Adult Male Dark Agouti (DA) rats, weighing 250g (Taconic A/S, Ejby, Denmark) were used and housed in a temperature-controlled environment with a 12-hour light/dark cycle with food and water *ad libitum*.

### Study design

Chronic renal failure (CRF) was induced by one-step 5/6 nephrectomy, as previously described [28]. In brief, a partial nephrectomy was performed at the left side, leaving 1/3 of the left kidney intact, followed by total nephrectomy on the right side. Rats were anesthetized with hypnorm-midazolam (Panum Institute, Copenhagen, Denmark), and were given carprofen (Rimadyl, Pfizer, Copenhagen, Denmark) subcutaneously as pain relief for the following three days. CRF-rats were fed a high-P diet throughout the study (0.9% Ca, 1.2% P, 600IU vitamin D per kg diet; Altromin Spezialfutter GmbH & Co., KG, Germany). After eight weeks of uremia, rats were treated with alfacalcidol (Leo Pharmaceutical, Copenhagen, Denmark) 80 ng intraperitoneally (ip) three times weekly to induce severe vascular calcification. After six weeks of alfacalcidol treatment, totalling 14 weeks of uremia, CRF rats were allocated to either high-dose BMP7 (CRF/BMP7, n = 6) or vehicle (CRF/Veh, n = 6) for 8 weeks or as aorta donor in a isogenic aorta transplantation (CRF/ATx). Recombinant human BMP7 was provided by Johnson and Johnson (Raritan, NJ, USA) to Keith Hruska. BMP7 and vehicle was given as a single ip administration once weekly, the BMP7 dose was 250μg/kg per animal weekly and the vehicle consisted of isotonic saline. At the time of allocation the alfacalcidol treatment was stopped. Normal age-matched DA rats were kept in parallel and fed a standard diet (0.9% Ca, 0.7% P, 600IU vitamin D per kg diet; Altromin Spezialfutter GmbH & Co., KG, Germany). Controls (n = 10) were left untreated and sacrificed after 22 weeks. In the ATx surgery the calcified abdominal aorta from the renal arteries to the bifurcation was transplanted orthotopically from uremic animals into healthy littermates. As surgical controls the aorta from a group of healthy animals was transplanted into healthy littermates (Ctrl/ATx). One rat from the CRF/ATx group and two rats from the Ctrl/ATx group died post-surgery. Recipients were sacrificed four weeks after the transplantation (CRF/ATx, n = 15 and Ctrl/ATx, n = 9). At the time of ATx, a section of the aorta graft just proximal to the renal arteries was removed and stored at -80°C to determine baseline Ca-content.

At sacrifice rats were anesthetized with pentobarbital (50 μg/kg ip; Nycomed-DAK, Copenhagen, Denmark) and eye blood was drawn. Kidney rudiments from uremic animals and corresponding segments from control animals were harvested for protein- and gene expression analysis. The right femur was harvested for gene expression analysis, removing all of the connective tissue and bone marrow, and the left femur was stored in 70% ethanol at 5°C for micro CT scan. The aorta was dissected from the bifurcation to the heart, and the connective tissue and blood was removed by gentle manipulation and rinsing with sterile saline. Tissue was snap frozen in liquid nitrogen and stored at -80°C to minimize RNA-degradation.

### Plasma biochemistry and aortic Ca-content

Uremia and the associated mineral metabolism disturbances were evaluated by measurement of plasma creatinine, urea, P, total Ca (TCa), ionized Ca (Ca^2+^), PTH and FGF23. Plasma P, urea, creatinine and TCa were analysed by Vitros 250 (Ortho-Clinical Diagnostics, Raritan, NJ, USA) and Ca^2+^ by ABL505 (Radiometer, Copenhagen, Denmark). Plasma intact FGF23 (iFGF23) was measured by a human FGF23 ELISA assay (Kainos Laboratories, Tokyo, Japan) measuring only full-length FGF23, with an intra-assay coefficient of variation of 2.5% and inter-assay coefficient of variation of 5% in our lab [29]. Plasma PTH was measured by a rat bioactive intact PTH ELISA assay (Immutopics, San Clemente, CA, USA) with an intra-assay variation of 4% and inter-assay variation of 9% in our lab [30].

Aortic calcium content was determined both in the proximal thoracic and distal abdominal aorta in the CRF/BMP7, CRF/Veh and control rats and in the proximal aorta in CRF/ATx and Ctrl/ATx rats by the o-cresolphthalein method and normalized to dry weight. A section of the thoracic and abdominal aorta was lyophilized for 24 hours in order to determine the dry-weight. After lyophilisation the aortic sections were decalcified in 1M HCl for 24 hours and the Ca-content of the supernatant was determined using a commercial assay (Sigma-Aldrich, St. Louis, USA).

### Quantitative RT-PCR

Thoracic aorta, femur and kidney tissue were manually grounded by mortar and pestle immersed in liquid nitrogen. Total RNA was extracted from the tissue-powder using a bullet blender and the EZNA RNA isolation kit (Omega Bio-tek, Norcross, GA). First-strand cDNA was synthesized from 0.3–1.5 μg of RNA with Superscript III cDNA kit (Invitrogen, Waltham, Ma). JumpStart (Sigma-Aldrich, St Louis, MO, USA) and Lightcycler 480 II (Roche, Basel, Switzerland) were used for quantitative real-time PCR, with a binding temperature of 59°C. The RNA integrity was determined in a representative number of samples of each tissue using the Agilent 2100 Bioanalyzer and software tool and PCR products for each primer set were confirmed on 2% agarose gels. The mRNA levels were normalized to the mean of reference genes and reference gene stability was tested using Genorm [31]. Primers are listed in the supplementary (S1 Table).

### Western blot

Protein was extracted from grounded kidney tissue in T-PER protein extraction reagent (Thermo Scientific, Rockford, IL) with Halt protease inhibitor cocktail (Thermo Scientific, Rockford, IL) using a Ultra-Turrax homogenizer. Protein concentration was determined by the BCA assay (Thermo Scientific, Rockford, IL). 30μg protein were run on Mini-Protean TGX stain-free precast gel (Bio-Rad, Germany) under reducing conditions and transferred onto nitrocellulose membranes (Bio-Rad, Germany). Membranes were blocked with 5% bovine serum albumin (BSA) (Roche, Germany) in phosphate buffered saline (PBS) with 0.05% tween. Primary and secondary antibodies were diluted in PBS with 3% BSA. The antibodies used were monoclonal anti-human Napi2a (1:1000) (NBP2-42216, Novusbio), polyclonal anti-human Napi2a (1:1000) (NBP2-13328, Novusbio), polyclonal anti-human Pit2 (1:500) (HPA026540, Atlas Antibodies), polyclonal anti-mouse klotho (1:2000) (ab154163, Abcam), polyclonal anti-human Park7 (1:1000000) (ab18257, Abcam). The secondary antibodies used were anti-rabbit (1:2000) (P0448, Dako) and anti-mouse (1:1000) (P0447, Dako). Western blot quantifications were performed with ImageJ.

### Micro CT

The left femur was dissected free of soft tissue, placed in 70% ethanol and stored at 4°C. High-resolution micro-computed tomography (microCT) was performed of the whole femur. CT images were acquired on an Inveon Multimodality PET/CT scanner (Siemens) with the following settings: 361 projections, 60 kV, 500 μA and 1300 ms exposure. Images were reconstructed with an isotropic voxel size of 32 μm. Image analysis was performed using the Inveon Software (Siemens). The length of femur was measured from the medial condyle to caput femoris. The distal femur growth plate was used as reference. The cortical bone cross-sectional area was measured approximately midshaft (10 mm proximal to the reference growth plate) by manually drawing the outer and inner perimeter, and then subtracting the ellipsoid areas. For analysis of trabecular bone, a region of interest (ROI) of 0.960 mm along the longitudinal direction was drawn manually starting at 2.080 mm proximal to the reference growth plate. The CT images of ROI were segmented into bone and marrow by a visually chosen fixed threshold for all groups. Bone morphometry was calculated using the Inveon Software based on the parallel plate model by Parfitt [32]. The following parameters were calculated: the ratio of total trabecular volume to total tissue volume (BV/TV), the ratio of trabecular bone surface to trabecular bone volume (BSa/BV), trabecular thickness (Tb/th, mm), trabecular number (Tb/no, 1/mm), trabecular spacing (Tb/sp, mm) and trabecular pattern factor (TPF). Bone mineral density (BMD) was calculated using phantom reference data. A standard bone phantom (Inveon, Siemens) was calibrated and applied to calculate the BMD of the trabecular bone.

### Ethics

The experimental studies were performed in accordance with the National Institutes of Health Guide for Use of Laboratory Animals and approved by the Animal Inspectorate, Denmark (Reference: 2012-DY-2934-00023). The animals were under daily supervision by animal caretakers and veterinarians from The Panum Institute, University of Copenhagen.

### Statistics

Data are presented as mean ± standard deviation (SD) or median and interquartile range (IQR). Unpaired two-tailed t-test was used to compare means between groups and paired two-tailed t-test was used to compare means within groups. Mann-Whitney U test was used to test for between group differences in PTH and microCT data. Wilcoxon matched-pairs signed rank test was used to test for within group differences in PTH. Correlations were calculated by Pearson’s correlation coefficient. The Holm-Bonferroni method was used to correct for multiple comparisons. P<0.05 was considered significant. Calculations were performed in Graphpad Prism 7.0.

## Results

### Effect of BMP7 administration on plasma phosphate in uremic rats

**Fig 1:**
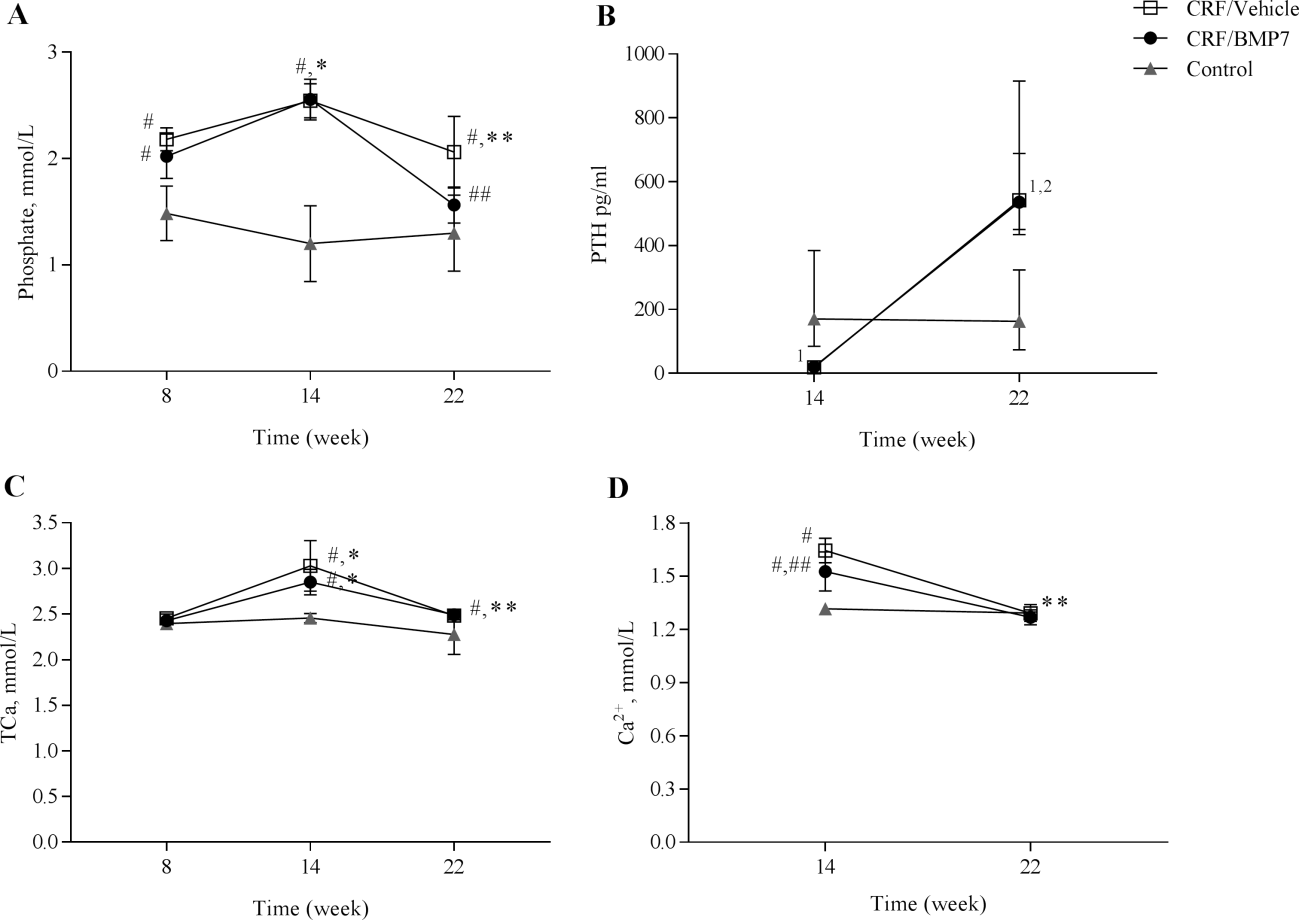
BMP7 had a plasma phosphate-lowering effect in uremic rats. The duration of uremia was 22 weeks. To induce severe vascular calcification the rats were treated with alfacalcidol from week 8 to 14. Then the CRF rats were treated with BMP7 (250 μg/kg/week) or vehicle for 8 weeks. (**A**) CRF rats had increased plasma phosphate (P) levels at 8 weeks, and alfacalcidol treatment resulted in a further increase at 14 weeks. At 22 weeks plasma P decreased in the vehicle treated rats to the level observed before alfacalcidol treatment. In the CRF/BMP7 group a significant further decrease in plasma P was observed, resulting in near normal plasma P, despite uremia and high P diet. (**B**) At 14 weeks plasma PTH was completely suppressed in CRF animals and increased to very high levels at 22 weeks similar in BMP7- and vehicle-treated animals. (**C-D**) Plasma total calcium (Tca) and Ca^2+^ were increased with a slight difference in plasma Ca^2+^ between the two uremic groups at 14 weeks. At 22 weeks plasma TCa and Ca^2+^ were normalized. Plasma P, TCa and Ca^2+^ is presented as mean ± SD and PTH as median and IQR, n = 6–10. *P<0.05 vs 8 weeks and ** P<0.05 vs 14 weeks by two-tailed paired t-test. ^#^P<0.05 vs control and ^#^P<0.05 vs vehicle by two-tailed unpaired t-test. ^1^P<0.005 vs control by Mann Whitney U-test. ^2^P<0.05 vs 14 weeks by Wilcoxon matched-pairs signed rank test.

Plasma biochemistry and body weight at 8, 14 and 22 weeks are presented in the supplementary (S2 Table). Plasma creatinine and urea were significantly elevated and comparable in the two uremic groups. At 22 weeks intact FGF23 (iFGF23) levels were elevated in the uremic animals (P<0.05 vs control). Plasma P, PTH, TCa and Ca^2+^ are presented in Fig 1. At 14 weeks plasma TCa and Ca^2+^ were elevated in the uremic animals after alfacalcidol treatment and dropped to normal at 22 weeks when the alfacalcidol treatment was stopped, Fig 1C and 1D. Consistently plasma PTH was completely suppressed in the uremic animals at 14 week and increased to above normal levels at 22 weeks when the alfacalcidol treatment was stopped, Fig 1B. There were no significant differences in plasma TCa or PTH between the two uremic groups at any time point, but a slight significant difference in plasma Ca^2+^ at 14 weeks (P<0.05). Plasma P levels were significantly elevated in the uremic animals at 8 weeks; 2.18±0.11mmol/L in the vehicle-allocated group and 2.02±0.21mmol/L in the BMP7-allocated group compared to that of controls 1.48±0.26mmol/L. At 14 weeks the plasma P levels further increased to 2.54±0.16mmol/L in the vehicle-allocated group and 2.56±0.19mmol/L in the BMP7-allocated group after alfacalcidol treatment. At 22 weeks plasma P decreased in the vehicle-treated group to 2.06±0.34mmol/L (P<0.05 vs 14 weeks) the same level as before the alfacalcidol treatment. In the BMP7-treated group, plasma P was reduced even further to a level of 1.56±0.17mmol/L (P<0.01 vs CRF/Veh), Fig 1A, and at 22 weeks plasma P in the BMP7-treated group was not significantly different from plasma P in the control group.

### Effect of BMP7 administration on deregulated gene expression in the calcified aorta

**Fig 2:**
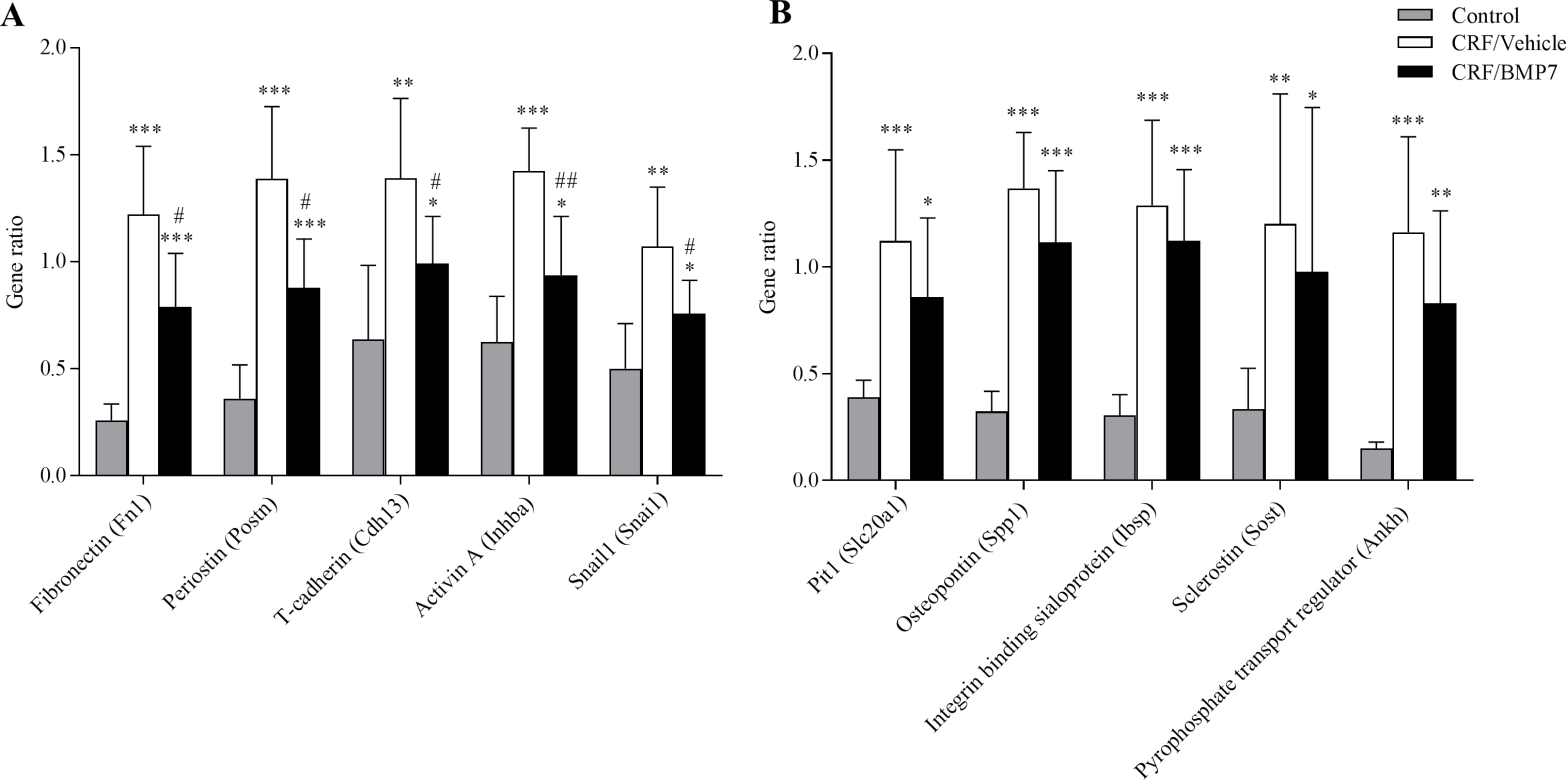
BMP7 ameliorated disturbed gene expression in the calcified aorta. The two figures depict expression levels of genes related to the calcification process. (**A**) In uremic rats the expression of genes involved in fibrosis and epithelial-to-mesenchymal transition (EMT) was strongly increased. The BMP7-treated group had significantly lower expression levels of these genes compared to vehicle. (**B**) There was a strong induction of genes related to extracellular matrix calcification and osteogenic transformation in the uremic rat aorta, but no significant differences were seen in the expression levels of these genes between the BMP7- and vehicle-treated uremic groups. Mean ± SD, n = 6–10. *P<0.05 vs control; **P<0.001; ***P<0.0001 vs control; ^#^P<0.05 vas vehicle; ^##^P = 0.006 vs vehicle by two-tailed unpaired t-test.

The effect of BMP7 treatment on the expression of genes related to the calcification process was examined by qPCR, Fig 2. In uremic rats an increase in the aortic expression of genes related to calcification, osteogenic transformation and fibrosis was observed. In the BMP7-treated group a significant lower expression of fibronectin, periostin, t-cadherin, inhibin-βa and snail1 was seen compared to the vehicle-treated rats (P<0.05 and P<0.01 vs CRF/vehicle), Fig 2A. In uremic rats aorta the expression of pit1, osteopontin, bone sialoprotein, sclerostin and the pyrophosphate transport regulator Ankh was increased, but no significant differences were seen in the expression levels of these genes between the BMP7- and vehicle-treated rats, Fig 2B.

### Effect of BMP7 administration and normalization of the uremic environment by aorta transplantation on the Ca-content of the calcified aorta

**Fig 3:**
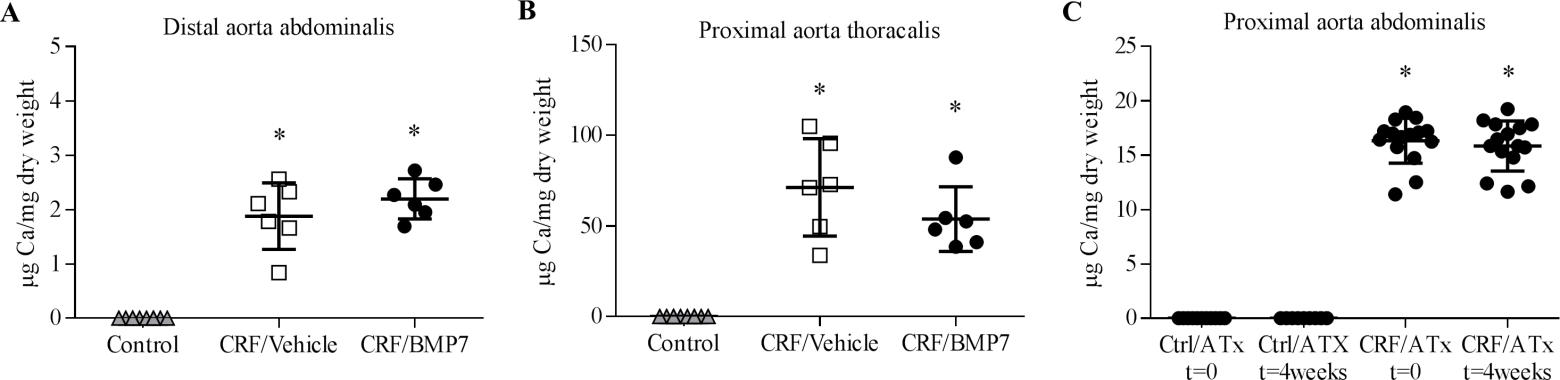
The effect of BMP7 treatment and normalisation of the uremic milieu on aortic Ca-content. Uremic alfalcalcidol-treated rats had significant calcification of both the distal abdominal, proximal abdominal and proximal thoracic aorta. (**A-B**) The Ca-content of the proximal thoracic aorta was 20–30 fold higher compared to the distal abdominal aorta. No differences were seen between BMP7- and vehicle-treated rats in the Ca-content in the established vascular calcification of either the abdominal or thoracic aorta. (**C**) Complete normalization of the uremic environment by aorta transplantation (ATx) did not change the Ca-content of the calcified aorta from CRF rats (CRF/ATx) and the surgery did not induce calcifications of the normal aorta from healthy animals (Ctrl/ATx). Mean ± SD, n = 6–15; *P<0.0001 vs control by two-tailed unpaired t-test.

Ca-content of both the distal abdominal and proximal thoracic aorta from uremic rats were significantly increased (P<0.0001 vs control), Fig 3. In the CRF/Vehicle rats Ca-content of the distal abdominal aorta was 1.9±0.6μg/mg, while the Ca-content of the proximal thoracic aorta was significantly higher 71±27μg/mg (P<0.002). Similar differences were seen in CRF/BMP7 rats; 2.2±0.4μg/mg in the distal abdominal aorta and 54±18μg/mg in the proximal thoracic aorta (P<0.001). The mean Ca-content of the thoracic aorta was not significantly lower in the BMP7-treated rats compared to the vehicle group. In the ATx group no differences were seen in the Ca-content of the transplanted calcified aorta at the time of ATx and 4 weeks after transplantation. No calcifications were seen in the normal aorta from healthy animals neither at the time of ATx or 4 weeks post-transplantation, Fig 3C.

In CRF rats strong positive correlations were found between the Ca-content of the thoracic aorta and gene expression of fibronectin (r = 0.93), osteopontin (r = 0.88), bone sialoprotein (r = 0.82), t-cadherin (r = 0.79), ankh (r = 0.79), inhibin-βa (r = 0.77) and Pit1 (r = 0.74), P<0.01, S1A–S1G Fig. No correlations were found with periostin, sclerostin and snail1, S1H–S1J Fig.

### Effect of BMP7 on kidney gene- and protein levels of P transporters

**Fig 4:**
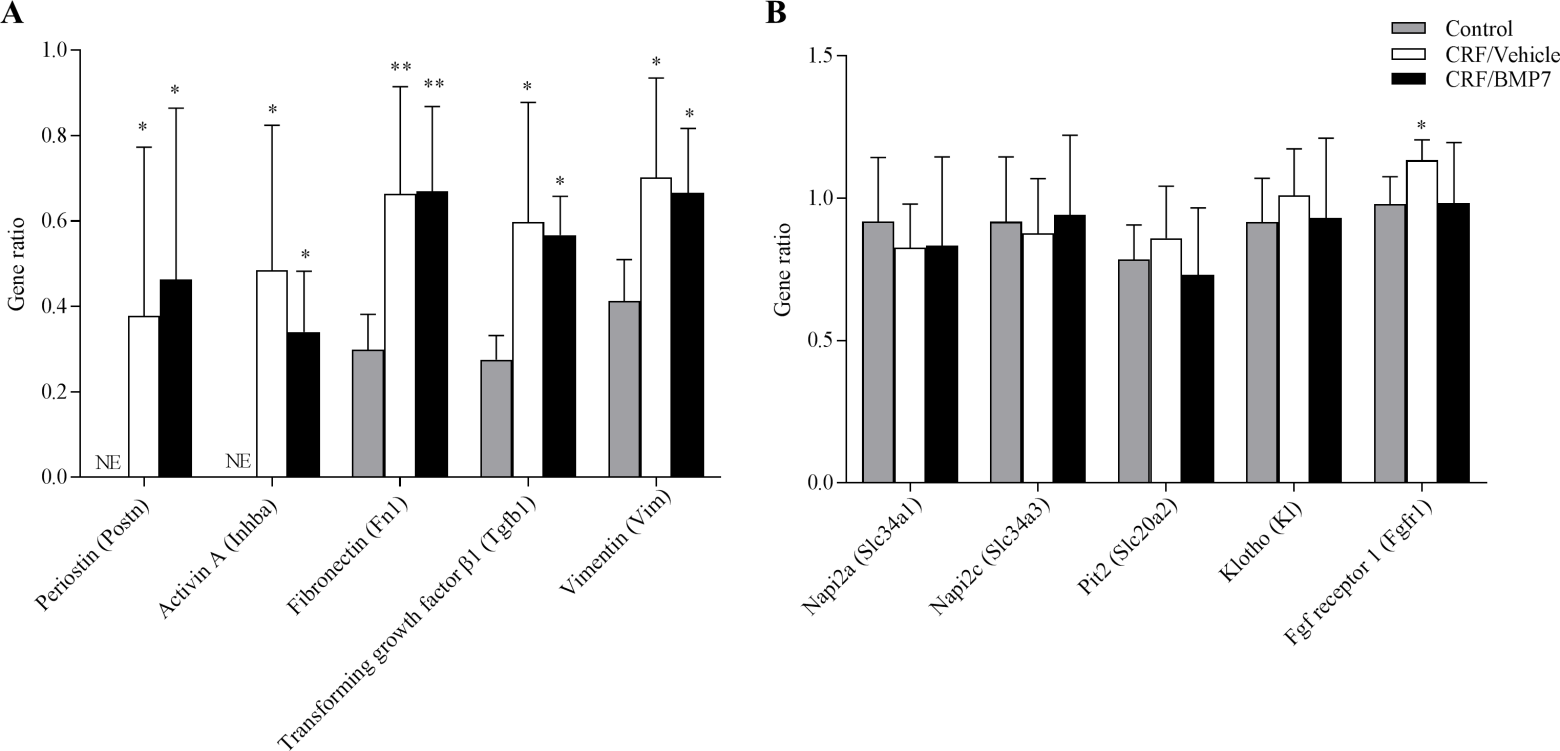
BMP7 treatment and kidney gene expression. (**A**) Uremia significantly increased the expression of genes related to fibrosis and EMT: Postn, Fn1, Tgfb1, Vim and Inhba. Postn (periostin) and Inhba (Activin A) were not expressed (NE) in kidneys from control rats, but significantly induced in uremic rats. No differences between CRF/BMP7 and CRF/Veh were seen in the expression levels of the examined genes. (**B**) No differences were seen between controls and CRF/Veh and CRF/BMP7 in the expression of the phosphate transporters Napi2a, Napi2c and Pit2 or in the FGF23 co-receptor, Klotho. In the CRF/Veh group there was a small increase in the expression of Fgfr1 and this increase was not seen in the CRF/BMP7 group. Mean ± SD, n = 6–10. *P<0.01; **P = 0.0007 vs control by two-tailed t-test.

The gene expression of markers of fibrosis including tgfβ1, vimentin and fibronectin were increased and gene expression of periostin and inhibin-βa were induced in kidneys from uremic animals, Fig 4A. No differences were seen in the expression of these genes between CRF/BMP7 and CRF/Veh. Furthermore, no difference was seen in the gene expression of the FGF23 co-receptor klotho between controls and CRF/Veh and CRF/BMP7. A significant and small increase in the gene expression of the FGF23 receptor fgfr1 was seen in the uremic vehicle-treated group (P<0.01 vs control), and this was normalized in the BMP7-treated group, Fig 4B.

Plasma P was normalized in the CRF/BMP7 group; therefore kidney gene expression and protein levels of P transporters were determined in order to examine whether this normalization of P was mediated by regulation of renal P excretion. No differences in the gene expression of the P transporters Napi2a, Napi2c and Pit2 were seen between the controls and the CRF/Veh and CRF/BMP7 groups, Fig 4B.

**Fig 5:**
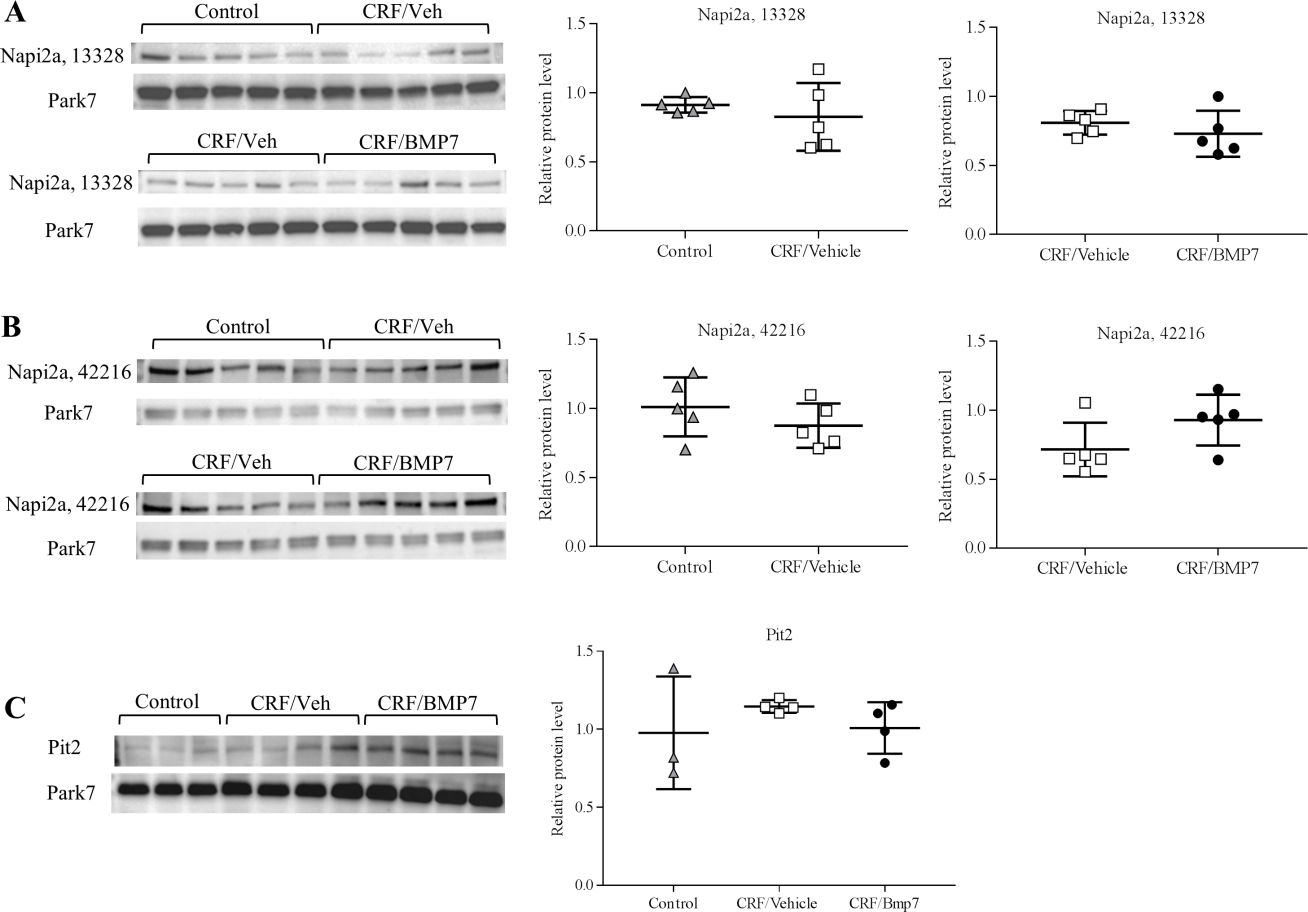
BMP7 treatment and the protein levels of phosphate transporters in the kidney. Protein levels of Napi2a and Pit2 in kidney tissue from control rats, uremic vehicle-treated rats (CRF/Veh) and uremic BMP7-treated rats (CRF/BMP7) were examined by Western blot. (**A-B**) No differences were seen in Napi2a protein levels between controls and CRF rats or between CRF/Veh and CRF/BMP7. To confirm the results blots were made with two different antibodies NBP2-13328 and NBP2-42216, targeting different peptide-sequences. (**C**) No difference was seen in Pit2 protein level between controls and CRF/vehicle and CRF/BMP7. Mean ± SD.

The results were confirmed by Western blot, where no differences were seen in protein levels of Napi2a and Pit2 between the vehicle- and BMP7-treated groups, Fig 5, and further confirmed by Western blots using two different antibodies. Klotho gene expression was not decreased in the uremic rats and similar results were found at the protein level by Western blot, S2A Fig. Uncropped blots are available in supplementary, S2 Fig.

### Effect of BMP7 on bone examined by qPCR and microCT

**Fig 6:**
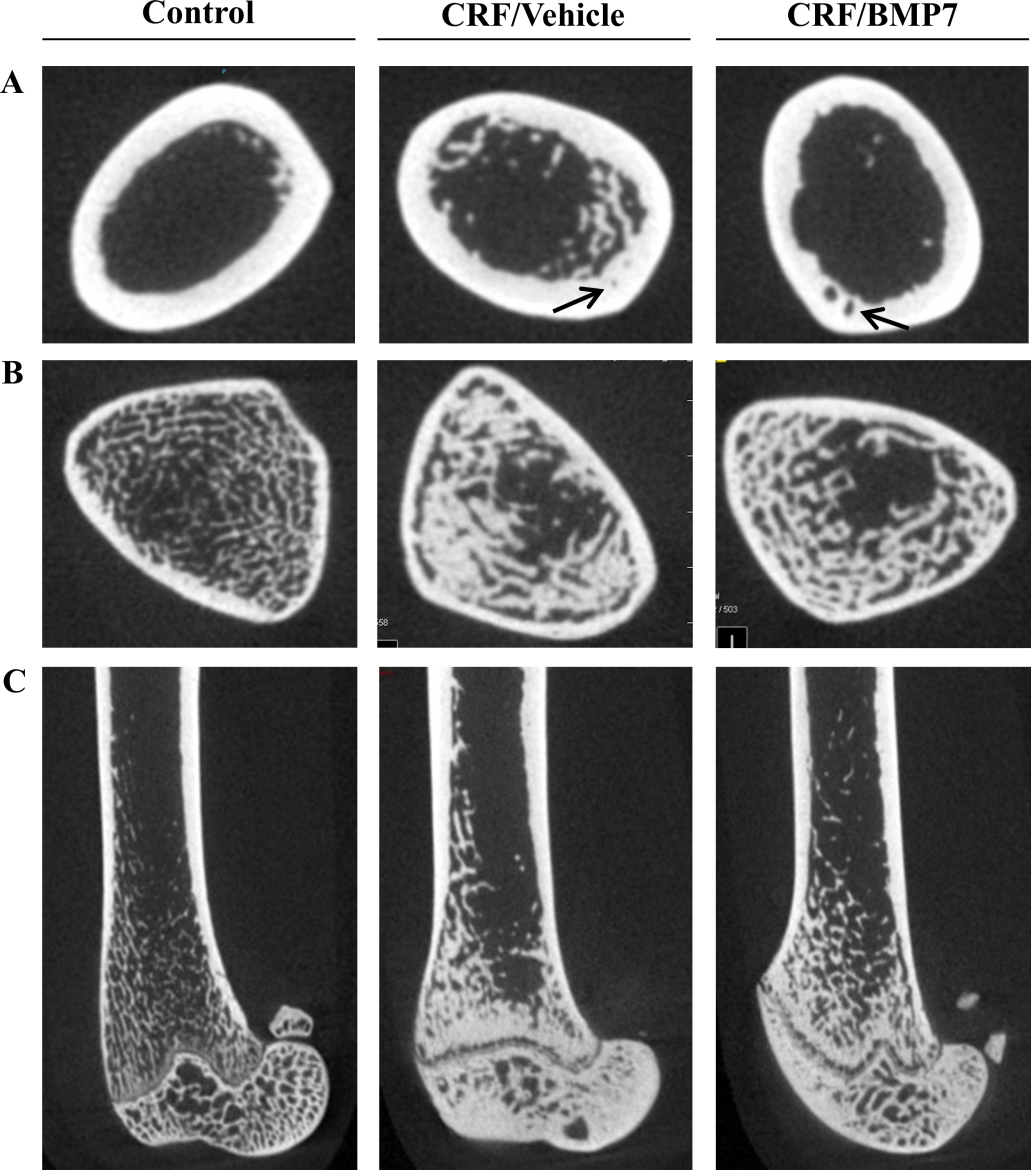
Effect of BMP7 on bone morphology visualized by microCT imaging. Representative pictures of distal femur from control rats, uremic vehicle treated rats (CRF/Veh) and uremic BMP7 treated rats (CRF/BMP7). (**A**) Cortical cross-sectional area midshaft. In comparison to control rats both uremic groups had thicker cortical bone with porosity, indicated by arrow. (**B**) Trabecular bone in region of interest (ROI). Uremic rats had increased trabecular thickness, greater trabecular spacing and less trabecular number. (**C**) Sagittal image of distal femur. No effect of BMP7 treatment was seen on bone microstructure.

Furthermore it was examined whether the P-lowering effect of BMP7 might be due to amelioration of renal osteodystrophy and hereby increased P uptake into bone. Therefore, the effect of BMP7 treatment of uremic rats on bone microstructure was examined by microCT, Fig 6, and bone gene expression was determined. Uremia and alfacalcidol treatment greatly increased bone mineral density (BMD) (P<0.001 vs control), and resulted in an increase in cortical thickness, a decrease in the number of trabeculae and greater trabecular spacing (P<0.05 and P<0.01 vs control), as presented in Table 1. BMP7 treatment however had no effect on bone microstructure. Bone gene expression data are available in the supplementary, S3 Fig. The expression of sclerostin was significantly decreased in the CRF/Veh and the CRF/BMP7 group compared to control (P<0.05). No significant differences were found between controls and the two uremic groups in the expression of periostin, Runx2, osteocalcin, Pit1, Pit2, osteopontin and cathepsin k.

**Table 1:** Effect of BMP7 on bone morphology and bone mineral density.

	Control Median [range]	CRF/Vehicle Median [range]	CRF/BMP7 Median [range]
**Cortical cross-sectional area**	0.16[0.15–0.18]	0.18*[0.17–0.19]	0.19*[0.18–0.19]
**BV/TV**	0.54[0.48–0.56]	0.48[0.28–0.67]	0.50[0.41–0.59]
**BSA/ BV**	17.8[16.7–20.1]	15.9[8.9–21.7]	15.0[11.4–18.3]
**Trabecular thickness (mm)**	0.11[0.10–0.12]	0.13[0.09–0.23]	0.14[0.11–0.17]
**Trabecular number**	4.81[4.23–5.04]	3.55**[2.95–3.76]	3.59**[3.20–4.19]
**Trabecular spacing(mm)**	0.11[0.09–0.12]	0.15*[0.11–0.23]	0.14*[0.12–0.15]
**BMD (mg/cc)**	1595[1589–1603]	2068**[2037–2096]	2060**[2058–2085]

Distal femur was analyzed by microCT technique. Uremic rats treated with alfacalcidol had increased cortical cross-sectional area midshaft with no difference between CRF/Vehicle and CRF/BMP7 groups. There were no differences between controls and CRF/Vehicle and CRF/BMP7 in bone volume over total volume (BV/TV) and bone surface area (BSA). CRF rats had increased trabecular thickness, a decrease in the number of trabeculae, greater trabecular spacing and markedly increased bone mineral density (BMD). Treatment with BMP7 had no significant impact on bone microstructure and density. *P<0.05; **P<0.01 with Mann Whitney U-test, n = 4.

## Discussion

In the present study it was examined whether BMP7 treatment might potentially be able to reverse established uremic vascular calcification (VC) as BMP7 previously has been shown to inhibit development and progression of this condition [26;27]. In a control model it was examined whether normalization of the uremic environment would reverse vascular calcification. This was done by performing an isogenic transplantation of a calcified aorta from uremic animals into healthy littermates. In the present model of uremic VC a combination of long-term uremia and high-dose alfacalcidol was used to induce severe calcification of the aorta. This experimental model of uremic calcified aorta had a massive increase in aortic expression of genes related to fibrosis (fibronectin, periostin) to epithelial-to-mesenchymal transition (EMT) (snail1) to ECM calcification (Pit1, osteopontin and bone sialoprotein) and osteogenic transformation (sclerostin and ankh) in agreement with our previous results [12]. Treatment with BMP7 significantly reduced aortic expression of profibrotic genes and genes related to EMT, while there were no significant changes in the expression of markers of ECM calcification. BMP7 is a member of the Tgfβ superfamily and an antagonist of Tgfβ signaling capable of inhibiting fibrosis in several organs including kidney, lung, liver and heart [33]. Increased Tgfβ signaling, altered ECM composition and EMT have been found to occur in the calcified vasculature indicating that fibrotic changes are important in uremic VC [34;35]. Fibronectin, periostin and snail1 are involved in fibrosis and EMT and they are all directly induced by Tgfβ signaling [34–37]. The expression of these genes was significantly reduced by BMP7 treatment. We have previously shown that the aortic expression of the t-cadherin gene increases in uremic VC [12] and an increased expression in atherosclerotic areas has also been shown by others [38]. Unlike other cadherins, t-cadherin is a glycosylphosphatidylinositol (GPI)-anchored protein which is suggested to be less involved in formation of adherence junctions, but could function as a signaling-receptor and a modulator of cell proliferation and migration [39]. In this respect an association between t-cadherin expression and SMC dedifferentiation has been suggested [40]. *In vitro*, Tgfβ-stimulated fibroblast-to-myofibroblast transition is associated with increased t-cadherin expression [41]. The decrease in t-cadherin expression by BMP7 treatment might potentially improve vascular function in uremia. Activin A is a dimer of two inhibin-βa subunits. It is another member of the Tgfβ superfamily with profibrotic capabilities and serum Activin A levels increases in CKD and in various tissues during inflammation and fibrosis [42]. In the present results BMP7 treatment significantly reduced the increased expression of inhibin-βa in the uremic aortas. Agapova et al have shown that a ligandtrap for the Activin receptor ActRIIA (RAP-011) decreased VC in the calcified aorta from high fat fed ldlr^-/-^ mouse with superimposed CKD [43]. It should however be noted, that the calcifications induced in that study were less severe and more punctuated than those reported in the present model [12].

BMP7 treatment did not have an effect on the total Ca-content of the calcified aorta despite the significant decrease in the expression of the profibrotic genes and normalization of plasma P levels. Neither had complete normalization of the uremic environment in the ATx control model any effect on the total Ca-content of the calcified aorta. No aortic calcifications were seen in healthy rats pre- or post-transplantation, confirming that the surgery did not induce calcifications. Spontaneous regression of calcitriol-induced vascular calcification has been shown in non-uremic rats after hesitation of calcitriol, but only after a very short calcitriol treatment period of 8 days [44]. Previously we have found significant differences between calcitriol-induced VC in uremic animals and in calcitriol treated non-uremic animals, but also clearly demonstrated that the major changes in gene expression profile in the present model were induced by uremia and not by calcitriol [12]. Furthermore, it has been proposed that uremia per se is a strong inhibitor of reversibility of VC [45]. In the present investigation, elimination of hyperphosphatemia or of the uremic milieu did not result in any regression of established VC. Lomashvili et al have studied the reversibility of calcitriol-induced uremic VC [46]. In parallel with the present ATx-model they performed an allogenic transplantation of a calcified aorta from uremic animals into healthy animals and examined the effect on VC. In the present investigation no change in the Ca-content of the transplanted aorta was found, while Lomashvili et al noted an immediate small decrease in the Ca-content, but afterwards no sign of active resorption was demonstrated. In the present study BMP7 treatment did not affect the Ca-content of the calcified aorta, which could be due to the severity of the calcifications induced. We found that BMP7 significantly reduced the aortic expression of genes related to fibrosis and EMT while genes linked to ECM calcification were not affected. Osteogenic transformation and subsequent calcification of the ECM might represent the end-stage of uremic vascular disease, whereas inflammation and/or fibrosis could be present at earlier stages and more readily treatable. Thus the present results might indicate that a necessary step in a potential reduction of established severe uremic VC could be repression of the extracellular matrix calcification in the vascular wall.

The BMP7 treatment resulted in a significant decrease in plasma P despite persistent uremia and a high P diet. A P-lowering effect of BMP7 in uremic animals is in agreement with a previous report [26]. It is highly interesting as high plasma P by itself is a vascular toxin in uremia and as the reduction during BMP7 treatment in the present study occurs despite no improvement in kidney function. The present study was unfortunately not designed to include measurements of urinary P. Therefore, in order to examine the potential mechanism of the P lowering effect of BMP7 we investigated whether this effect was due to increased P excretion by examining kidney gene expression levels of the phosphate transporters Napi2a, Napi2c and Pit2 and protein levels of Napi2a and Pit2. No differences were found in either gene expression or protein levels. It is interesting that the uremic animals did not have a decrease in Napi2a, Napi2c and Klotho levels, as we have previously reported this in a uremic model on a high P diet without active vitamin D [47]. This difference is probably an effect of the long-term alfacalcidol treatment. Both the Napi2a and the Klotho gene contain a functional vitamin D responsive element, and it has previously been reported, that active vitamin D can induce Napi2a and Klotho expression [48;49].

Another possible explanation for the reduced plasma P levels during BMP7 treatment might be that P was built into bone. Long bones from uremic and control animals were therefore examined by microCT and qPCR. Results obtained by microCT showed that our model of long-term uremia and treatment with alfacalcidol had increased bone mineral density, increased cortical thickness and a decreased number of trabeculae with greater spacing. These disturbances were probably induced by the combination of the mineralization effect of active vitamin D and the complete suppression of PTH resulting in a heavily mineralized adynamic bone. Consistent with the increased bone mass on microCT, gene expression levels of sclerostin decreased in the uremic animals. Sclerostin inhibits bone formation by inhibiting Wnt-signalling, and disorders with decreased sclerostin activity are characterized by increased bone growth [50]. BMP7 has previously been shown to improve both high- and low turnover bone diseases [24–26]. In the present investigation however no effect of BMP7 treatment was found on bone as examined by microCT. In the previous studies bones were examined by histology and histomorphometry, reporting no differences in trabecular number or trabecular spacing in uremic animal similar to the present results, but with an effect on osteoblast number, mineralizing surface and bone formation rates [25;26]. We cannot exclude a potential effect of BMP7 on these parameters in our study. Furthermore, the possibility of an effect of BMP7 on the intestinal phosphate absorption was not included in the present investigation and should be addressed in future studies. Alternatively, BMP7 might have an effect on the size of the exchangeable phosphate pool [51]. An exchangeable pool has been demonstrated for other ions such as ionized calcium and magnesium [52;53] independent of bone turnover.

In summary, treatment with BMP7 resulted in significant suppression of Tgfβ-induced expression of profibrotic genes in the aorta of a uremic experimental model with severe vascular calcification induced. Treatment with BMP7 resulted in normalization of plasma phosphate which potentially may have beneficial effects on the vasculature in uremia independent of BMP7. The total Ca-content of the uremic calcified vasculature was however not significantly improved by BMP7. Normalization of the uremic environment for four weeks as achieved by performing an isogenic transplantation of a calcified aorta into a healthy littermate neither changed the Ca-content of the uremic aorta. The present results highlight the importance of preventing the development of vascular calcification in CKD. Once established, vascular calcification persists even in the setting when hyperphosphatemia or the uremic milieu is abolished.

## Supporting information

S1 Fig Correlations: Ca-content of thoracic aorta vs gene expression levels. Correlations between Ca-content of the thoracic aorta and expression levels of genes induced in the uremic, calcified aorta. Strong correlations were seen between the Ca-content of the thoracic aorta and the expression of Fn1 and Spp1, moderate correlations were seen with the expression levels of Cdh13, Ankh, Inhba, Pit1 and Isbp, whereas only a weak correlation weas seen with Postn and no correlations were seen with Sost and Inhba. **○** CRF/Vehicle ● CRF/BMP7.
(TIF)

S2 Fig Uncropped western blot. Protein levels of phosphate transporters Napi2a and Pit2 and the FGF23 co-receptor Klotho was examined by western blot. Uremia and alfacalcidol treatment resulted in a small increase in klotho protein levels (**A**) and BMP7 had no effect on klotho levels (**B**). No differences were seen in Napi2a protein levels between the controls and CRF/vehicle and CRF/BMP7 examined with two different antibodies (**A-D**). No differences were seen in Pit2 protein levels between controls and CRF/vehicle and CRF/BMP7 (**E**). Mean±SD, *P<0.05, n = 5.
(TIF)

S3 Fig Bone gene expression. Mean expression levels of markers of bone formation, ECM mineralization and osteoblast activity. There was a significant decrease in the expression of sclerostin in the uremic alfacalcidol treated animals. BMP7 treatment did not change the expression of sclerostin. No other significant differences were seen. Mean±SD, n = 6–10. *P<0.05 vs control.
(TIF)

S1 Table Primer sequences. (PDF)

S2 Table Plasma biochemistry and bodyweight. *P<0.05 vs 8 weeks, **P<0.05 vs 14 weeks, ^#^P<0.05 vs control, ^##^P<0.05 vs vehicle.
(PDF)
