# Supplementary figures and images for: Exogenous BMP7 in aortae of rats with chronic uremia ameliorates expression of profibrotic genes, but does not reverse established vascular calcification
Source: PLoS One. 2018 Jan 5;13(1):e0190820. doi: 10.1371/journal.pone.0190820 (PMC5755916; doi:10.1371/journal.pone.0190820)

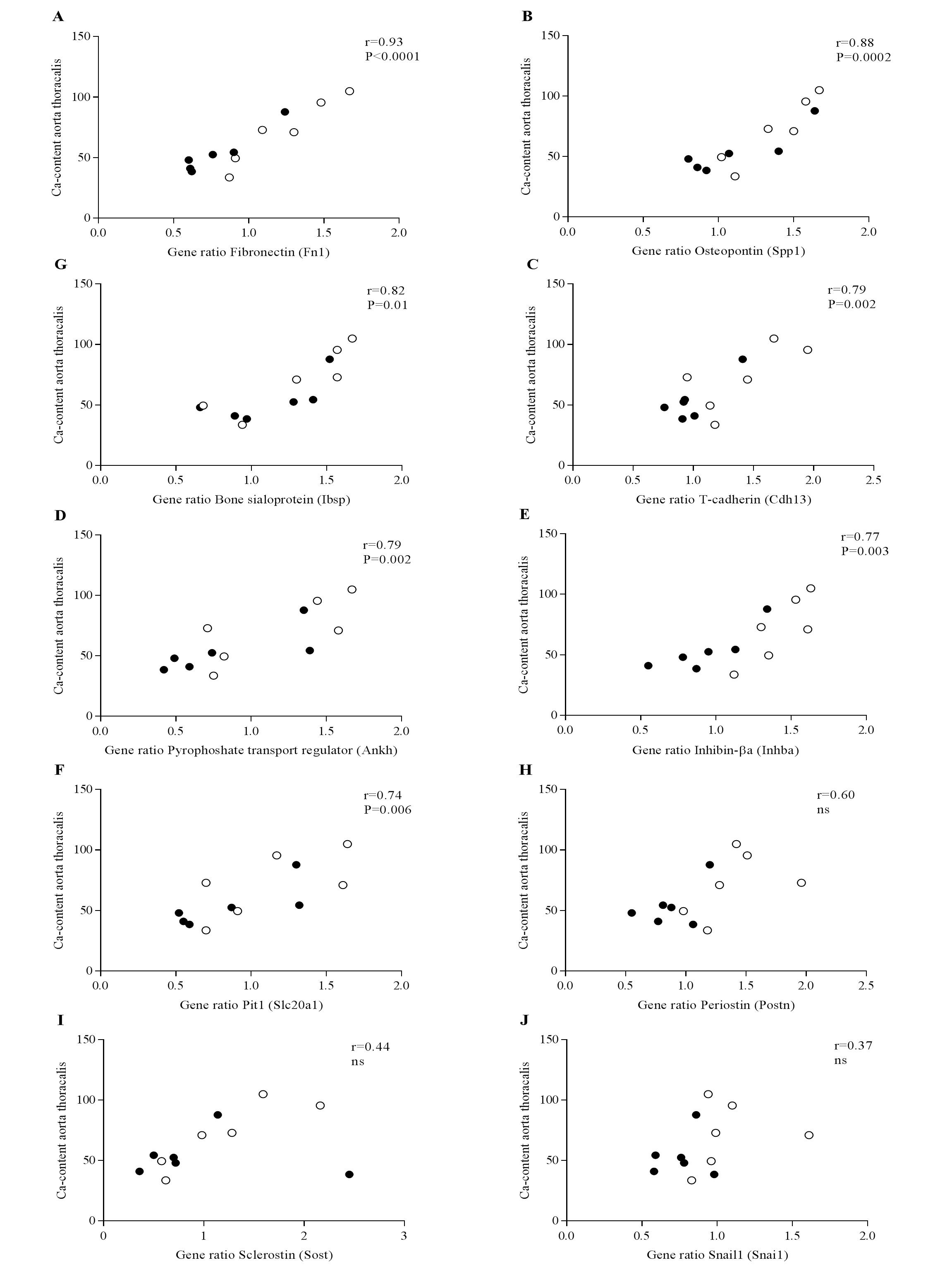

Supplement: S1 Fig — Correlations between Ca-content of the thoracic aorta and expression levels of genes induced in the uremic, calcified aorta. Strong correlations were seen between the Ca-content of the thoracic aorta and the expression of Fn1 and Spp1, moderate correlations were seen with the expression levels of Cdh13, Ankh, Inhba, Pit1 and Isbp, whereas only a weak correlation weas seen with Postn and no correlations were seen with Sost and Inhba. ○ CRF/Vehicle ● CRF/BMP7. (TIF) [file pone.0190820.s001.tif]

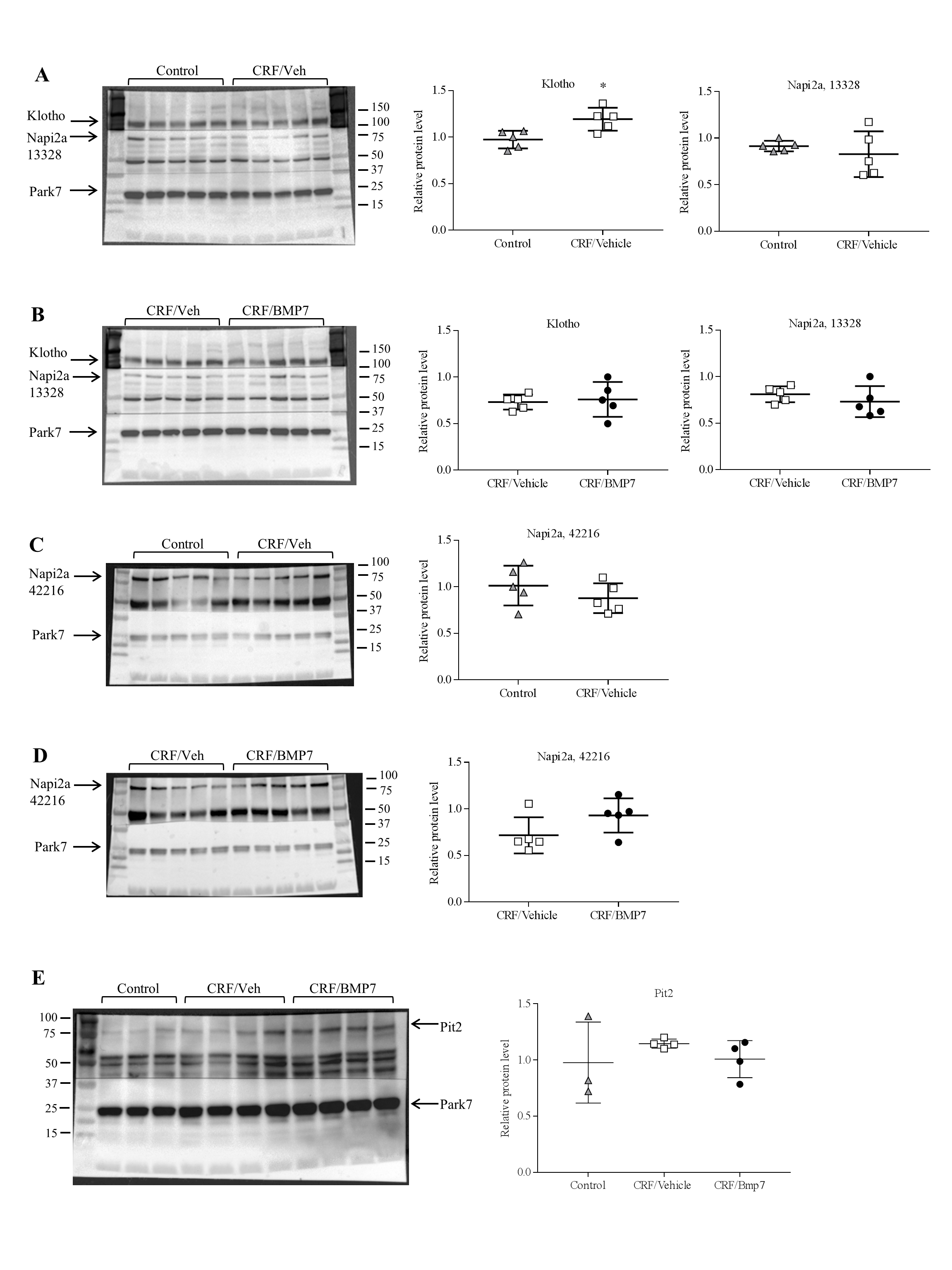

Supplement: S2 Fig — Protein levels of phosphate transporters Napi2a and Pit2 and the FGF23 co-receptor Klotho was examined by western blot. Uremia and alfacalcidol treatment resulted in a small increase in klotho protein levels (A) and BMP7 had no effect on klotho levels (B). No differences were seen in Napi2a protein levels between the controls and CRF/vehicle and CRF/BMP7 examined with two different antibodies (A-D). No differences were seen in Pit2 protein levels between controls and CRF/vehicle and CRF/BMP7 (E). Mean±SD, *P<0.05, n = 5. (TIF) [file pone.0190820.s002.tif]

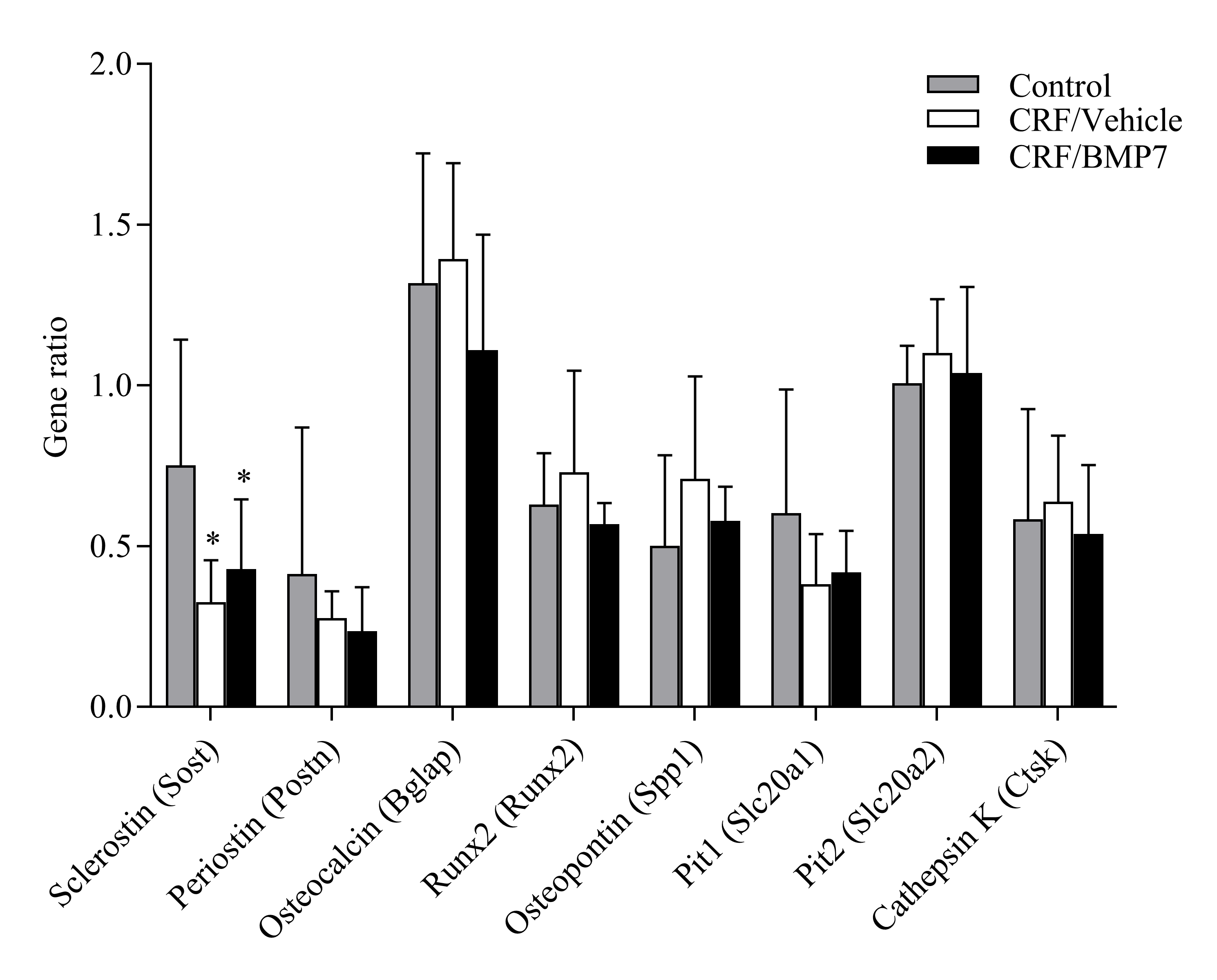

Supplement: S3 Fig — Mean expression levels of markers of bone formation, ECM mineralization and osteoblast activity. There was a significant decrease in the expression of sclerostin in the uremic alfacalcidol treated animals. BMP7 treatment did not change the expression of sclerostin. No other significant differences were seen. Mean±SD, n = 6–10. *P<0.05 vs control. (TIF) [file pone.0190820.s003.tif]
