# Supplementary material for: Exogenous BMP7 in aortae of rats with chronic uremia ameliorates expression of profibrotic genes, but does not reverse established vascular calcification
Source: PLoS One. 2018 Jan 5;13(1):e0190820. doi: 10.1371/journal.pone.0190820 (PMC5755916; doi:10.1371/journal.pone.0190820)
Supplement: S1 Table — (PDF) [file pone.0190820.s004.pdf]

Gene symbol	Name		Sequence
**Actb**	Actin, beta	F	**5'**-GAACCCTAAGGCCAACCGTGAA-**3'**
		R	**5'**-GCGCGTAACCCTCATAGATG-**3'**
**Ankh**	ANKH inorganic pyrophosphate transport regulator	F	**5'**-GGGCGACGCAACCATAAGAAAGAT-**3'**
		R	**5'**-TGTGGGCCGAGGTGACCGTGTTGT-**3'**
**Bglap**	Osteocalcin	F	**5'**-CCGTTTAGGGCATGTGTTGC-**3'**
		R	**5'**-CCGTCCATACTTTCGAGGCA-**3'**
**Cdh13**	T-cadherin	F	**5'**-GACTGGCACCGGGACTCTGATGA-**3'**
		R	**5'**-GAGGGCGTGGGTGTTGTTGATTTT-**3'**
**Ctsk**	Cathepsin K	F	**5'**-GGGAGACATGACCAGCGAAGAA-**3'**
		R	**5'**-CACACTGGCCCTGGTTTTTGAC-**3'**
**Eef1a1**	Eukaryotic translation elongation factor 1 alpha 1	F	**5'**-CATCGTCGTAATTGGACACG-**3'**
		R	**5'**-CACGCTAGCTTTCAGTTTG-**3'**
**Fgfr1**	Fibroblast growth factor receptor 1	F	**5'**-CCAGATCCTGAAGACTGCTGGAGT-**3'**
		R	**5'**-CTCTTCCAGGGCTTCCAGAACGGT-**3'**
**Fn1**	Fibronectin 1	F	**5'**-GGGCGAAGGCAATGGGCGTATCA-**3'**
		R	**5'**-CTGGGGGTGGGTCTGGGGTTGGTA-**3'**
**Gapdh**	Glyceraldehyde-3-phosphate dehydrogenase	F	**5'**-CAAGATGGTGAAGGTCGGTGTG-**3'**
		R	**5'**-TGAGGTCAATGAAGGGGTCGTT-**3'**
**Hprt**	Hypoxanthine phosphoribosyltransferase 1	F	**5'**-CCCAGCGTCGTGATTAGTGA-**3'**
		R	**5'**-CCAAATCTTCAGCATAATGATTAGGTAT-**3'**
**Ibsp**	Bone sialoprotein	F	**5'**-TACCGGCCACGCTACTTTCT-**3'**
		R	**5'**-GCGCAGCTAACTCCAACTTTC-**3'**
**Inhba**	Inhibin beta A subunit	F	**5'**-AGTGGGGGAAAACGGGTATGTGGA-**3'**
		R	**5'**-CCTGTTGGCCTTGGGGACTTTCA-**3'**
**Kl**	Klotho	F	**5'**-GGCCGACCATTTCAGGGATTAC-**3'**
		R	**5'**-TGGGGCGGAAGGAGGTGTTGTAGA-**3'**
**Postn**	Periostin	F	**5'**-GAACCCGGAGTCACCAACATC-**3'**
		R	**5'**-TGCTGCCACGAACGAACTTA-**3'**
**Rplp0**	Ribosomal protein lateral stalk subunit P0 (Arbp)	F	**5'**-AAAGGGTCCTGGCTTTGTCT-**3'**
		R	**5'**-GCAAATGCAGATGGATCG-**3'**
**Rpl13a**	Ribosomal protein L13A	F	**5'**-CCCTCCACCCTATGACAAGA-**3'**
		R	**5'**-CCTTTTCCTTCCGTTTCTCC-**3'**
**Runx2**	Runt-related transcription factor 2	F	**5'**-TCCATTCCACCACGCCGCTGTCTT-**3'**
		R	**5'**-GCCTGGGAACTGCCTGGGGTCTGA-**3'**
**Slc20a1**	Pit1	F	**5'**-CCGAACTGATAAAAATTGTGATGT-**3'**
		R	**5'**-GTTTGTCAAAGCCCAGCAATGGTG-**3'**
**Slc20a2**	Pit2	F	**5'**-CTGGCGTGGTGACCCTGAGACA-**3'**
		R	**5'**-AGACCGCTGAGCCGACCATT-**3'**
**Slc34a1**	Napi2a	F	**5'**-CTGGCGGGCTCCCTGGTGGTC-**3'**
		R	**5'**-AGGGGGCTGGGAAGTCTGTGTTGA-**3'**
**Slc34a3**	Napi2c	F	**5'**-TCTTCGCAGTTCAGGTTG-**3'**
		R	**5'**-GTGAGTAGTAAGTAGACAATGG-**3'**
**Snai1**	Snail1	F	**5'**-ATATTGTAATAAGGAGTACCTCA-**3'**
		R	**5'**-AGGCCTGACACTGGTATCTCTTC-**3'**
**Sost**	Sclerostin	F	**5'**-GCCTCCTCAGGAACTAGAGAAC-**3'**
		R	**5'**-TACTCGGACACGTCTTTGGTG-**3'**
**Spp1**	Osteopontin	F	**5'**-CCGAGGTGATAGCTTGGCTT-**3'**
		R	**5'**-TCGGACTCCTGGCTCTTCAT-**3'**
**Tgfb1**	Transforming growth factor, beta 1	F	**5'**-TCCACGTGGAAATCAATGGGATCA-**3'**
		R	**5'**-CAGTTCTTCTCTGTGGAGCTGAA-**3'**
**Vim**	Vimentin	F	**5'**-CGTGATGTCCGCCAGCAGTAT-**3'**
		R	**5'**-TGGCGCAGGGCATCGTTGTT-**3'**

S1 Table. Primer sequences.
