# Supplementary material for: Exogenous BMP7 in aortae of rats with chronic uremia ameliorates expression of profibrotic genes, but does not reverse established vascular calcification
Source: PLoS One. 2018 Jan 5;13(1):e0190820. doi: 10.1371/journal.pone.0190820 (PMC5755916; doi:10.1371/journal.pone.0190820)
Supplement: S2 Table — *P<0.05 vs 8 weeks, **P<0.05 vs 14 weeks, #P<0.05 vs control, ##P<0.05 vs vehicle. (PDF) [file pone.0190820.s005.pdf]

	Week	Control (n=10)	CRF/Vehicle (n=6)	CRF/BMP7 (n=6)
**Body weight, g** Mean ± SD	8	231 ± 17	258 ± 16 $^{\#}$	224 ± 12 $^{\#\#}$
	14	250 ± 23	190 ± 14 $^{\#}$, *	186 ± 23 $^{\#}$, *
	22	284 ± 17 *, **	275 ± 12 **	284 ± 10 *, **
**Urea, mmol/L** Mean ± SD	8	5.9 ± 0.6	11.6 ± 1.7 $^{\#}$	9.8 ± 1.8 $^{\#}$
	14	5.8 ± 0.5	9.8 ± 1.2 $^{\#}$, *	9.9 ± 0.7 $^{\#}$
	22	6.5 ± 0.6 *, **	12.2 ± 3.2 $^{\#}$, **	12.0 ± 2.8 $^{\#}$, *
**Creatinine, µmol/L** Mean ± SD	8	33 ± 4	48 ± 4 $^{\#}$	42 ± 5 $^{\#}$
	14	39 ± 3 *	53 ± 8 $^{\#}$	48 ± 5 $^{\#}$, *
	22	33 ± 6	57 ± 6 $^{\#}$, *	52 ± 9 $^{\#}$, *
**Phosphate, mmol/L** Mean ± SD	8	1.48 ± 0.26	2.18 ± 0.11 $^{\#}$	2.02 ± 0.21 $^{\#}$
	14	1.20 ± 0.36	2.54 ± 0.16 $^{\#}$, *	2.56 ± 0.19 $^{\#}$, *
	22	1.30 ± 0.36	2.06 ± 0.34 $^{\#}$, **	1.56 ± 0.17 $^{\#\#}$
**Total Ca, mmol/L** Mean ± SD	8	2.40 ± 0.06	2.46 ± 0.05	2.43 ± 0.02
	14	2.46 ± 0.05	3.03 ± 0.28 $^{\#}$, *	2.85 ± 0.14 $^{\#}$, *
	22	2.28 ± 0.22	2.48 ± 0.05 $^{\#}$, **	2.49 ± 0.05 $^{\#}$, **
**Ca^{2+}, mmol/L** Mean ± SD	8	-	-	-
	14	1.32 ± 0.03	1.65 ± 0.07 $^{\#}$	1.53 ± 0.11 $^{\#}$, $^{\#\#}$
	22	1.29 ± 0.05	1.29 ± 0.01 **	1.27 ± 0.04 **
**PTH, pg/mL** Median [IQR]	8	-	-	-
	14	170 [85-385]	19 [18-21] [1]	21 [17-30] [1]
	22	163 [74-324]	542 [435-689] [1], [2]	536 [451-915] [1], [2]
**Intact FGF23, pg/mL** Mean ± SD	8	-	-	-
	14	-	-	-
	22	313 ± 77	1816 ± 741 $^{\#}$	1205 ± 543 $^{\#}$

S2 Table. Plasma biochemistry and bodyweight. *P<0.05 vs 8 weeks and **P<0.05 vs 14 weeks by two-tailed paired t-test, $^{\#}$P<0.05 vs control and $^{\#\#}$P<0.05 vs vehicle by two-tailed unpaired t-test. [1]P<0.005 vs control by Mann Whitney U-test. [2]P<0.05 vs 14 weeks by Wilcoxon matched-pairs signed rank test.
